# Dry needling as an adjunct treatment to multimodal rehabilitation protocol following rotator cuff repair surgery: a preliminary, randomized sham-controlled trial

**DOI:** 10.1186/s12998-024-00555-y

**Published:** 2024-12-05

**Authors:** Faeze Naseri, Mehdi Dadgoo, Mohammadreza Pourahmadi, Morteza Nakhaei Amroodi, Shirin Azizi, Amirhossein Shamsi

**Affiliations:** 1 Iranian Center of Excellence in Physiotherapy, Rehabilitation Research Center, Department of Physiotherapy, School of Rehabilitation Sciences, Iran University of Medical Sciences, Tehran, Iran; 2 Bone and Joint Reconstruction Research Center, Department of Orthopedics, School of Medicine, Iran University of Medical Sciences, Tehran, Iran; 3 Department of Physiotherapy, School of Rehabilitation, Tehran University of Medical Sciences, Tehran, Iran

**Keywords:** Dry needling, Postoperative shoulder Pain, Trigger point, Rotator cuff tear, Rotator Cuff repair, Shoulder rehabilitation

## Abstract

**Background:**

Rotator cuff repair (RCR) is one of the most prevalent procedures to manage rotator cuff tears (RCT). Postoperative shoulder pain is a common complication following RCR and may be aggravated by activation of myofascial trigger points (MTrP) associated with the injury to the soft tissues surrounding the surgical incision. This study aimed to describe a preliminary, randomized, sham-controlled trial to evaluate the effectiveness of implementing 4 sessions of myofascial trigger point dry needling (MTrP-DN) as a muscle treatment approach along with 10 sessions of multimodal rehabilitation protocol (MRh) consisting of therapeutic exercise, manual therapy, and electrotherapy on postoperative shoulder pain, range of motion (ROM), strength, and functional outcome scores for patients following RCR surgery.

**Methods:**

Forty-six patients aged 40–75 following RCR surgery were recruited and randomly allocated into 2 groups: (1) MTrP-DN plus MRh (experimental group), and (2) sham dry needling (S-DN) plus MRh (control group). This trial had a 4-week intervention period. The primary outcome was the Numeric Pain Rating Scale (NPRS) for postoperative shoulder pain. Secondary outcomes were the Shoulder Pain and Disability Index (SPADI), ROM, and strength. The mentioned outcomes were measured at baseline and week 4. In the current study, adverse events were recorded as well.

**Results:**

No statistically significant differences were observed between groups when adding MTrP-DN to MRh for postoperative shoulder pain after 4 weeks of intervention (mean difference 0.32, [95% CI -0.41,1.05], *p* = 0.37). However, this trial found a small effect size for postoperative shoulder pain. No significant between-group differences were detected in any of the secondary outcomes (*p* > 0.05) either. We found significant within-group changes in all studied outcome measures. (*p* < 0.001). This study also reported minor adverse events. following the needling approach.

**Conclusion:**

The lack of statistically significant differences in the outcomes and small clinical significance in shoulder pain highlights the complexity of pain management, suggesting that alternative methodologies may be needed for meaningful clinical benefits. Future studies should consider different control groups, long-term follow ups, larger sample sizes, and more MTrP-DN sessions to better understand their potential impact.

**Trial registration:**

This trial was registered at (https://www.irct.ir), (IRCT20211005052677N1) on 19/02/2022.

## Background

Shoulder pain is an important medical issue with significant health-care costs and an extensive impact on the affected individuals’ well-being including absence from work and disability [1]. Rotator cuff tear (RCT) is one of the most prevalent causes of shoulder pain and dysfunction arising from trauma or age- related degenerative changes [2]. Furthermore, RCTs are more common among individuals over 60 and may appear as either symptomatic or asymptomatic [2].

The treatment chosen for patients with RCT varies based on the size of the tear and patient-reported symptoms [2]. According to recent studies, Rotator cuff repair (RCR) surgery is recommended for patients with a full-thickness tear who are under the age of 65 and have repairable tendons with low risk of tendon retraction, or for those with a partial-thickness tear whose symptoms have not improved following non-operative treatment procedures [2]. RCR has demonstrated good long-term clinical results with more than 90% satisfaction in 10-year follow-up [3].

Following RCR and a period of relative postoperative immobilization, patients may suffer from shoulder pain, ROM restriction, weakness and loss of upper extremity function [4]. Moreover, RCR surgery as a mechanical trauma to the shoulder muscles can activate myofascial trigger points (MTrPs) [5]. Recent studies have provided evidence for the presence of MTrPs in patients with a history of rotator cuff pathology and the high rate of MTrPs in rotator cuff muscles [6, 7]. Moreover, considerable evidence suggests that MTrPs are caused by soft tissue lesions, such as rotator cuff disease [5, 6]. Rather than being the primary cause of shoulder pain, MTrP is often associated with other shoulder lesions, such as rotator cuff disease, which can overlap with the symptoms of shoulder lesions and exacerbate the pain [5, 6]. MTrP is defined as a hypersensitive area within a taut band of a skeletal muscle that might be painful on compression, stretching, or muscle contraction and may produce a referred pain pattern [5]. MTrPs are classified as active or latent [5]. When MTrPs are active, they may cause spontaneous pain and the elicited referred pain resembles the symptoms that the patient experiences [5]. If MTrPs are latent, they do not produce any spontaneous symptoms, and the elicited referred pain fails to produce the patient’s symptoms [5]. RCR, as a mechanical trauma to the soft tissues surrounding the shoulder joint, may convert latent MTrPs to active MTrPs by increasing the release of inflammatory mediators and aggravate postoperative shoulder pain and dysfunction [5, 8]. Also, neuro-motor abnormalities of shoulder girdle muscles brought on by MTrPs may exacerbate symptoms and prolong the recovery time [9].

A proper rehabilitation protocol following RCR is necessary for patients to restore their upper extremity function [4]. The rehabilitation program following RCR aims to relieve pain, restore passive and active ROM, strengthen shoulder girdle muscles, prevent shoulder joint stiffness and muscle atrophy, and return to daily activities [4]. Based on a Cochrane review by Green et al., combining therapeutic exercise, manual therapy, and electrotherapy as a comprehensive rehabilitation protocol has demonstrated to be beneficial for patients suffering from rotator cuff disease [10]. Furthermore, according to the possibility of activation of MTrPs following surgery, adding a muscle treatment approach to the routine rehabilitation programs after RCR may help patients recover faster [11].

Myofascial trigger point dry needling (MTrP-DN) is one of the main muscle treatment approaches to manage MTrPs pain [11]. It is an invasive technique which includes inserting an acupuncture-like needle into the involved muscles and is regularly used by physiotherapists all over the world along with other therapeutic interventions [11]. Recent studies have provided evidence for short and medium term effects of MTrP-DN for shoulder and neck pain compared to sham dry needling (S-DN) [12]. A randomized clinical trial by Arias-Buría et al. showed that adding a single session of MTrP-DN to a rehabilitation protocol for patients with a history of shoulder surgery may assist with faster recovery of function, although no significant differences were found in postoperative shoulder pain or ROM [13]. Moreover, a randomized controlled trial by Halle et al. found that using MTrP-DN for shoulder girdle muscles in standard rehabilitation care plans did not improve postoperative shoulder pain, ROM, or functional outcomes [14]. Consequently, based on the controversial findings of the previous studies regarding the effects of MTrP-DN for patients with postoperative shoulder pain and dysfunction [13, 14], and due to the lack of standardization in MTrP-DN dosage for patients following shoulder surgery [15], and also, based on our knowledge, the lack of study on usage of MTrP-DN in patients following RCR, this trial was designed to compare the effects of implementing 4 sessions of MTrP-DN in a multimodal rehabilitation protocol (MRh) on postoperative shoulder pain, ROM, strength and Shoulder Pain and Disability Index (SPADI) in patients who had undergone RCR to a control group that received S-DN.

### Objectives and hypotheses

This trial’s primary objective was to determine the effects of MTrP-DN compared to S-DN in a MRh on postoperative shoulder pain for patients following RCR. The secondary objectives were to determine the effects of MTrP-DN compared to S-DN in the MRh in both groups on ROM, shoulder muscles’ strength, and SPADI. We hypothesized that the patients who received MTrP-DN as part of their MRh would exhibit greater improvements than those who received S-DN instead.

## Methods

### Trial design

This study was designed as a preliminary, single center, superiority, randomized, sham-controlled trial with a parallel group of 46 patients. The allocation ratio was 1:1. This study follows the CONSORT guidelines, checklist and flowchart. You can find more details regarding the protocol of this trial at 10.1186/s12891-023-06269-1 [16].

### Participants

This study was conducted in Shafa Yahyaian Hospital’s physiotherapy clinic, Tehran, Iran. The participants were patients with a history of rotator cuff tendon tear that had undergone open RCR surgery in Shafa Yahyaian Hospital. The open RCR procedure involves a 5-cm incision with a sabercut approach and detachment of the deltoid muscle to reattach the rotator cuff tendons to greater tuberosity [4]. The number of involved muscles, size of tears (partial or full thickness tears), and tear retraction levels were different in each patient, and all operations were performed by a skilled surgeon (MNA) with more than 15 years of experience in shoulder and elbow surgery using the same procedure for all RCT patients. The inclusion and exclusion criteria are shown in Table 1.

**Table 1:** Inclusion and exclusion criteria

Inclusion criteria	Exclusion criteria
1) Status following RCR surgery. 2) Age range between 40 and 75 years. 3) Suffering from shoulder pain after 5 weeks of RCR surgery. 4) Palpable active MTrPs in the shoulder girdle muscles.	1) Needle phobia 2) History of coagulation disorders and consumption of anticoagulants. 3) Surgical history of the head and neck. 4) Radiculopathy and myelopathy disorders. 5) Pregnancy 6) No active MTrPs were discovered.

### Interventions

The participants were randomly assigned into experimental or control groups, which received either MRh with MTrP-DN (n = 23) or S-DN (n = 23). Both groups were treated by a physiotherapist (FN) with more than 4 years of clinical experience. The best available rehabilitation protocol following RCR was provided to both groups 3 times a week, for a total of 10 sessions. In addition, each session lasted for one hour. The inclusion of MTrP-DN extended the duration of each session by an additional 20 min, resulting in a total session length of an hour and twenty minutes. You can find more details on session distribution and exercise instructions in the protocol of this trial [16].

#### Multimodal rehabilitation protocol

There were 3 parts to each treatment session as described below:

Participants in both groups received conventional Transcutaneous Electrical Nerve Stimulation (TENS) at the beginning of each session on the operated shoulder for 20 min at a frequency of 120 HZ and a duration of 50 µs [17].

Following electrotherapy, participants received passive glenohumeral and scapulothoracic joints mobilization. Passive glenohumeral mobilization techniques (distraction, inferior glide, posterior glide and anterior glide) were applied for 2 sets of 20 repetitions and passive scapulothoracic mobilization techniques (inferior glide, superior glide, medial glide, lateral glide, upward rotation, downward rotation, depression and retraction) were applied for 10 repetitions of each movement [18–21].

Finally, the participants were prescribed with a set of therapeutic exercises based on the exercise progression protocols described by Giangarra et al. [22]. These exercises were divided into ROM and strengthening exercises and progressed over the course of 4 weeks. The first week was dedicated to passive ROM exercises (pendulum exercises, forward bow and passive ROM exercises with the physiotherapist for flexion, abduction, internal rotation and external rotation) which progressed to active assisted ROM exercises (wand exercises for flexion, internal rotation, extension and external rotation in supine position, and wash the table) in the second week [23]. In the third week, active ROM exercises (pulley exercises in 3 directions including flexion, internal rotation and external rotation, wall slide, standing arm elevation in scapular plane and standing shoulder flexion) were added to the patients’ program [23]. Lastly, participants started strengthening exercises (horizontal shoulder abduction and extension with low resistance TheraBands) in the fourth week [23]. The exercise dosages were based on previous studies’ recommendations, which were 3 times a day for 3 sets and 10 repetitions of each exercise for both groups at home and one time during their visit at the clinic [22, 23].

#### Trigger point diagnosis

MTrP diagnosis was performed by a physiotherapist (FN) with more than 4 years of experience in the management of MTrPs, using the following criteria: (1) Palpable taut band of a skeletal muscle that contains a hyperalgesic nodule, (2) Visible local twitch response to snapping palpation, or (3) Reproduction of referred pain brought on by palpating the hyperalgesic spot [5]. These standards have demonstrated good inter-examiner reliability (κ, 0.84–0.88) when utilized by an expert clinician [24].

Participants were examined for active MTrPs through flat palpation of the hyperirritable nodules in the shoulder girdle muscles including upper, lower, and middle trapezius, deltoid, levator scapula, supraspinatus, infraspinatus, subscapularis, teres minor, teres major, rhomboids, and pectoralis major based on the possibility of mechanical trauma to the rotator cuff and deltoid muscles during surgery, as well as the possibility of mechanical overload to the shoulder girdle muscles as a result of the period of relative shoulder immobilization, sling wear, and scapular dyskinesis [5, 13, 25].

#### Experimental group

Prior to MTrP-DN, participants were placed in a proper position, and the exact location of the MTrP was marked and cleaned with alcohol. Following the trigger point diagnosis, participants received MTrP-DN with disposable and sterile acupuncture needles (0.3 × 50 mm, EACU™ Acupuncture Needles) that were inserted into the hypersensitive nodules of MTrP using guide tubes. The “fast in and fast out” technique presented by Hong was applied in this trial [26]. According to Hong’s technique, once the first local twitch response is obtained, the needle is moved up and down for approximately 25 to 30 s until local twitch response exhaustion and patient’s tolerance limit are reached [11, 26]. The method of MTrP-DN was based on the approaches presented by Dommerholt and Fernandez de-las-penas [11]. MTrP-DN was used for a total of 3 muscles per session because patients with postoperative shoulder pain may exhibit MTrPs in more than 3 muscles and may experience muscle soreness following MTrP-DN, which may prevent them from consistently performing their exercises sets at the clinic or home [13, 27]. The needles remained in the affected muscles for 20 min [28].

#### Control group

Participants in the control group received S-DN. Based on Braithwaite’s study, the “penetrating S-DN” that is demonstrated as a “participant blinding strategy” was also utilized in this trial [29]. Accordingly, after the patients were placed in a proper position, and the exact location of MTrP was cleaned with alcohol, the acupuncture needle was inserted subcutaneously using the index finger to tap the needle into the epidermis until it was capable of supporting its own weight [29, 30]. There was no manipulation or local twitch response and the needle remained on the skin for 20 min [28]. It should be noted that the position of the patients, type and size of the needle was the same in both experimental and control groups.

### Outcomes

The outcome measurements were performed by a blind assessor (SA; who was unaware of group allocations) before the commencement of the first treatment session and after the end of the tenth session (2 evaluations in total).

#### Primary outcome measure

##### Resting pain

In the current study, resting pain assessed by the Numeric Pain Rating Scale (NPRS) was the main outcome measure. NPRS is a subjective scale that is scored from 0 to 10. The participants were asked to rate their level of pain over the previous 24 h, with the range being “0” for no pain to “10” for the worst pain imaginable [31]. According to recent studies, the NPRS is a valid and reliable scale for patients with shoulder pain [32].

#### Secondary outcome measures

##### Passive and active ROM

Passive and active shoulder ROM were the secondary outcome measures in this trial that were measured using a standard 18 cm plastic goniometer. The measured shoulder movements comprise the following: flexion, abduction, internal rotation, and external rotation to the pain-free end range. Passive flexion and abduction were measured while the patients were in a supine position with arms at their sides during which the distal end of the humerus was grasped by the assessor and moved to the pain-free end range [33]. Passive external rotation was measured in a supine position while the shoulder was 90° abducted, the elbow 90° flexed, and the forearm in mid-position. Then, the assessor moved the shoulder to the limit of pain-free external rotation [33]. Passive internal rotation was measured in a prone position with the shoulder abducted to 90°, the elbow flexed to 90° and the forearm in mid-position. Then, the assessor moved the shoulder to the limit of pain-free internal rotation [33]. Active ROM measurements were assessed through the same manner and positions however the participants were asked to actively perform the required movements to the pain-free end range [33].

##### Shoulder pain and disability index (SPADI) persian version

This 13 item questionnaire was utilized to evaluate the patients’ pain and disability during the activities of daily living [34]. According to recent studies, SPADI is a valid and reliable tool to be used for the Persian-speaking population [35].

##### Strength in shoulder movements

The isometric strength was measured at 45° of flexion and abduction in the sitting position using a handheld dynamometer (SF-50 Digital Force Gauge Dynamometer), during which the participants were asked to hold each isometric contraction for 5 s and repeat each movement for 3 times with proper rests between each trial. The average of these 3 trials was then used for data analysis [36].

### Sample size

The sample size was calculated using G-Power software (version 3.1.9.4). F-test as a family test and Analysis of Covariance (ANCOVA) as a statistical test were used. Minimal Clinically Important Difference (MCID) in postoperative shoulder pain as measured by NPRS was reported to be 2.17 [31]. In line with a previous study by Halle et al., the pooled standard deviation and the effect size were estimated to be 1.79 and 0.66, respectively, for NPRS after MTrP-DN following shoulder stabilization surgery [14]. Power and α error values were set to 95% and 0.05, respectively. Prior to the trial’s commencement, the required sample size was estimated at 34, and finally, 46 subjects were enrolled in order to account for a possible 30% dropout rate due to the invasive nature of the study.

#### Sequence generation

Prior to the randomization process, recruitment was carried out by receiving a list of patients who had undergone RCR at Shafa Yahyaian hospital and conducting a telephone interview with them. Following the interview, the patients who met the inclusion criteria were invited to the hospital’s physiotherapy clinic for MTrP diagnosis. Then, the eligible participants were assigned to either MTrP-DN or S-DN with an allocation ratio of 1:1. The randomization process was performed by computer using the blocked randomization method with 4 character blocks containing letters A or B (letter A indicating ‘experimental group’ and letter B indicating ‘control group’). The randomization schedule was transferred into written instructions and placed in sequentially numbered, opaque, and sealed envelopes. The numbered envelopes were distributed to each participant according to their ordinal number upon admission to the study after the preliminary assessment. The therapist used the information in each patient’s envelope to plan the intervention. To avoid data contamination, patients were advised not to give the assessor their allocation information after being placed in the target group. Assignment and enrollment were performed by the hospital’s secretary and sequence generation was performed by a person who was outside the research team.

### Blinding and concealment

Due to the nature of the study, it was impossible to maintain the blinding of the principal physiotherapist, although the participants and the outcome assessor were blinded to the group allocation. Additionally, group allocation was concealed from study personnel during the screening process before randomization.

### Statistical methods

Data were analyzed using STATA software version 16 (Stata Corp LLC). Continuous data were reported in mean **±** standard deviation (SD) and categorical data were reported in frequency counts (%). Data normality was checked for all continuous variables using the Shapiro-Wilk test, histogram, and skewness and kurtosis. The paired t-test was used for the within-group analysis. The analysis of variance and covariance (ANOVA/ANCOVA) was used to determine between group differences of all continuous data while the pre-intervention values of outcomes were included as a covariate and groups as a factor (one factor, one covariate; primary analysis). Also, in other analysis models called sensitivity analysis, we selected age as another covariate (one factor, two covariates) and gender as another factor (two factor, two covariate) since mean differences (MD) of age and gender between groups were considerable (more than 0.2 × SD in age and more than 10% difference in gender between groups) [37–39]. The level of significance was set at 0.05. In addition, the point estimates of effects were presented as MD with a 95% confidence interval (CI) and standardized mean differences (SMD) with 95% CI, analyzed using Cohen’s d. According to the newly presented definition, the Cohen’s d effect size can be divided as follows: from 0.01 to 0.19: very small, from 0.2 to 0.49: small, from 0.5 to 0.79: medium, from 0.8 to 1.19: large, from 1.2 to 1.99 very large, more than 2: huge [40]. Cohen’s d effect size was also used to evaluate three analysis models and assess the effects of confounder/covariable on results. Changes of Cohen’s d more than 10% between three analytic models were considered significant. The intention to treat (ITT) was used to analyze primary and secondary outcomes. The simple imputation (Forward fill) was used to account for missing data and dropouts. All participants were included in the analysis.

## Results

**Fig. 1:**
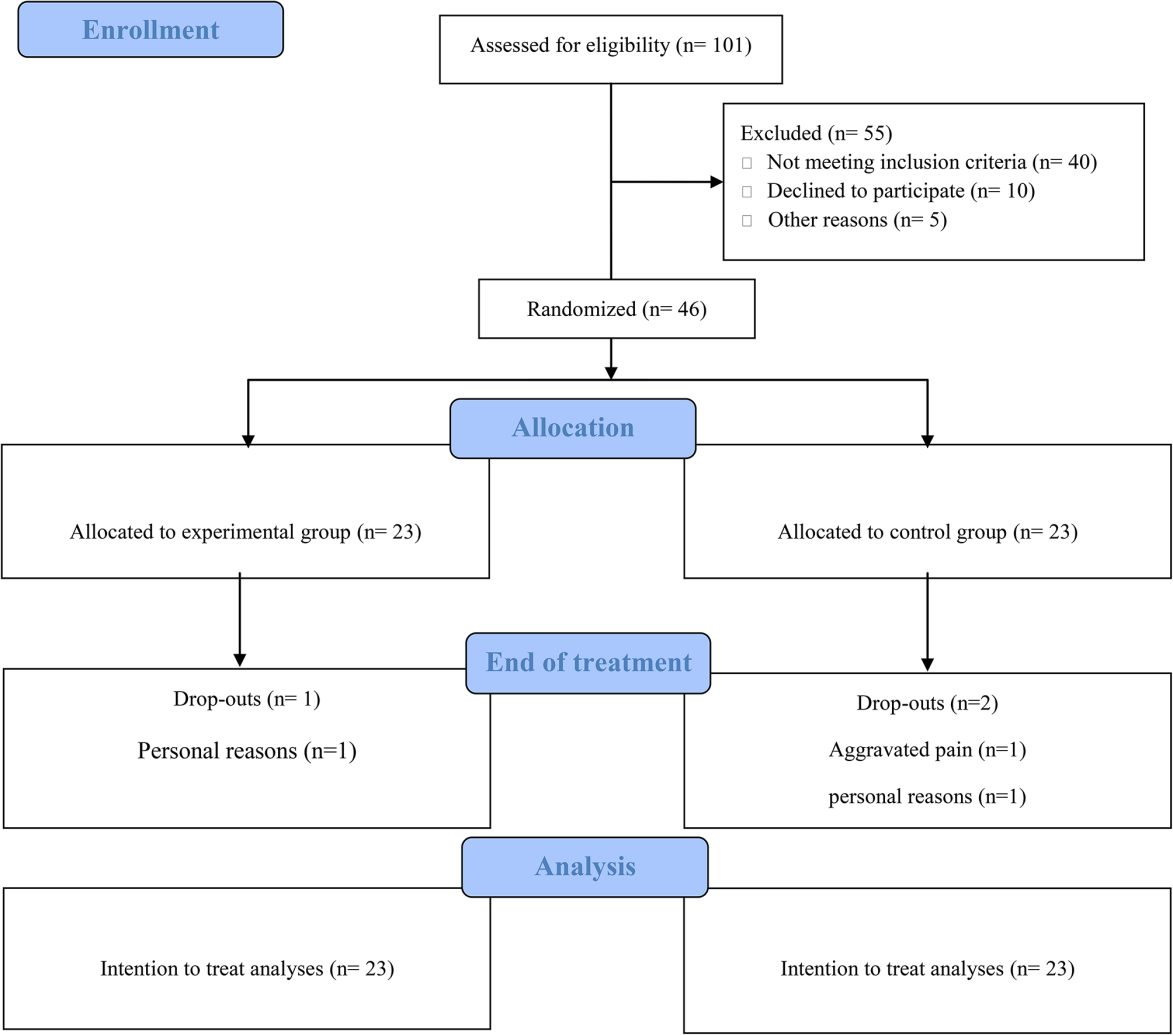
CONSORT flow-chart related to the stages of the study

We screened 101 patients following RCR surgery by telephone interviews, and 53 patients did not match the inclusion criteria. 48 patients were invited to the hospital for trigger point diagnosis, and 46 were eventually added to the trial. 3 participants dropped out of the study (2 in the control group and 1 in the experimental group; Fig. 1). The participant in the experimental group dropped out after a session of therapy due to personal reasons and the participants in the control group dropped out due to aggravated pain and personal reasons after 2 sessions. Finally, 43 participants (21 in the control group and 22 in the experimental group) completed the rehabilitation protocol. The participants’ baseline demographic characteristics are provided in Table 2.

### Distribution of MTrPs

Table 3 provides information regarding the distribution of MTrPs that is well-balanced across groups.

**Table 2:** Demographic and clinical characteristics of the experimental group and control group at the baseline after RCR surgery

Variables	Experimental group (*n* = 23)	Control group (*n* = 23)
Age (years)	58.04 (6.65)	61.69 (8.4)
Gender, n (%)		
Male Female	11 (48%) 12 (52%)	6 (26%) 17 (74%)
Height (cm)	166.91 (9.25)	163.08 (10.25)
Weight (kg)	75.21 (12.33)	76.21 (18.25)
Time from surgery (week)	6.91 (1.64)	7.17 (1.52)
Analgesic usage	7 (30%)	8 (34%)
Number of active MTrPs	2.6 (1.33)	2.04 (1.18)
Operated shoulder, n (%)		
Right Left	17 (74%) 6 (26%)	17 (74%) 6 (26%)
Dominant hand, n (%)		
Right Left	22 (96%) 1 (4%)	22 (96%) 1 (4%)
IPAQ-SF (total score)	564.88 (274.95)	511.88 (253.81)

Data are mean (SD) unless indicated

IPAQ-SF, international physical activity questionnaire- short form

^*^ All of the participants’ characteristics are statistically similar between groups based on independent student t-test and chi-square test

^*^ Independent student t-test

^**^ Chi-square test

**Table 3:** Distribution of MTrP in patients following RCR

Muscles		Deltoid	Upper trapez	Middle trapez	Lower trapez	Levator scapula	Supraspinatus	Infraspinatus	Subscapularis	Teres minor	Teres major	Rhomboids	Pectoralis major
**Number reported**	**Control**	21	13	3	3	5	1	2	1	2	1	4	3
	**Experimental**	20	13	3	3	8	2	3	2	2	2	5	2

### Primary outcome measure

In the ANCOVA with adjusting pain to its pre-intervention values (model 1 primary analysis), we didn’t find any statistically significant between-group differences and the effect size was “small” (MD: 0.32 [-0.41-1.0], SMD: 0.23 [-0.35 to0.81]; Table 4). Additionally, in our study, we evaluated the clinical significance of the intervention by assessing patients’ changes in NPRS met or exceeded the MCID of 2.17 points [31]. Accordingly, 60% of patients in the experimental group (14/23) and 52% of patients in the control group (12/23) experienced a change in NPRS that met or exceeded the MCID. Moreover, the changes of Cohen’s d between three analytical models were not significant (less than 10%). Accordingly, even differences in baseline measurements, age, and gender couldn’t affect the findings of our study (Table 4). The results of within-group analysis revealed that both the experimental and control groups improved in pain, as measured by NPRS (*p* < 0.001; Table 4).

### Secondary outcome measures

In the ANCOVA with adjusting secondary outcomes to their pre-intervention values (primary analysis), the mean A-FLX P-FLX, A-IR, P-IR weren’t statistically different between groups in all analysis models (*p* > 0.05; Table 4). Furthermore, the changes of Cohen’s d between three analytical models were not significant (less than 10%; Table 4). Accordingly, differences in the baseline measurements, age, and gender couldn’t affect the findings of the mentioned outcomes. The mean A-ABD, P-ABD, A-ER, P-ER, S-FLX, S-ABD, SPADI weren’t statistically different in all analysis models (*p* > 0.05; Table 4). Although in these outcome measures, the changes of Cohen’s d between three analytical models were significant (more than 10%; Table 4). Based on these results, A-FLX, P-FLX, A-IR, and SPADI showed “small” effect sizes, but other secondary outcome measures did not show statistical or clinical improvements and showed trivial effect sizes (Table 4). In addition, we found significant within-group changes in all studied outcome measures (SPADI, ROM, strength, *p* < 0.001; Table 4).

**Table 4:** Values of NPRS, ROM, strength, and SPADI for MTrP-DN group and S-DN group

Outcome	Experimental (*n* = 23)			Control (*n* = 23)			(*P* value)^**^	MD [95% CI]	SMD [95% CI]
	Baseline^*^	4 weeks^*^	*P* value	Baseline^*^	4 weeks^*^	*P* value			
NPRS	5.3 (1.49)	2.17 (1.41)	< 0.001	4.98 (1.26)	2.31 (1.48)	< 0.001	(0.379) (0.387) (0.475)	0.32 [-0.41-1.05] ^a^ 0.33 [-0.43-1.09] ^b^ 0.28 [-0.51-1.09] ^c^	0.23 [-0.35 to 0.81] ^a^ 0.24 [-0.34 to 0.82] ^b^ 0.21 [-0.37 to 0.79] ^c^
A-FLX	111.74 (32.6)	153.83 (12.72)	< 0.001	111.52 (31.8)	148.52 (19.62)	< 0.001	(0.262) (0.299) (0.352)	-5.26 [-14.61-4.08] ^a^ -5.02 [-14.74-4.68] ^b^ -4.76 [-14.98-5.46] ^c^	-0.32 [-0.9 to 0.26] ^a^ -0.31 [-0.89 to 0.27] ^b^ -0.29 [-0.87 to 0.29] ^c^
A-ABD	77.74 (26.85)	115.83 (27.06)	< 0.001	87.35 (25.05)	124.61 (32.56)	< 0.001	(0.809) (0.904) (0.955)	1.69 [-12.39-15.77] ^a^ 0.87 [-13.74-15.49] ^b^ -0.42 [-15.67-14.81] ^c^	0.06 [-0.52 to 0.63] ^a^ 0.03 [-0.55 to 0.6] ^b^ -0.01 [-0.59 to 0.56] ^c^
A-ER	44.26 (14.41)	66.43 (10.27)	< 0.001	47.48 (20.04)	66.74 (19.14)	< 0.001	(0.620) (0.542) (0.409)	-1.65 [-8.35-5.04] ^a^ -2.11 [-9.06-4.83] ^b^ -2.97 [-10.19-4.28] ^c^	-0.11 [-0.69 to 0.47] ^a^ -0.14 [-0.72 to 0.44] ^b^ -0.2 [-0.78 to 0.38] ^c^
A-IR	35.26 (9.66)	53.13 (12.68)	< 0.001	39.09 (8.3)	52.78 (11.61)	< 0.001	(0.295) (0.362) (0.363)	-3.24 [-9.44-2.94] ^a^ -2.93 [-9.36-3.49] ^b^ -3.04 [-9.74-3.64] ^c^	-0.27 [-0.85 to 0.37] ^a^ -0.25[-0.83 to 0.33] ^b^ -0.25 [-0.83 to 0.32] ^c^
P-FLX	128.91 (26.63)	164.87 (11)	< 0.001	129.17 (26.6)	159.48 (20.39)	< 0.001	(0.233) (0.198) (0.234)	-5.45 [-14.55-3.64] ^a^ -6.09 [-15.51-3.31] ^b^ -5.9 [-15.79-3.98] ^c^	-0.34 [-0.92 to 0.24] ^a^ -0.38 [-0.96 to 0.2] ^b^ -0.36 [-0.95 to 0.22] ^c^
P-ABD	94.52 (21.41)	130.96 (24.04)	< 0.001	101.96 (25.03)	135.57 (30.96)	< 0.001	(0.899) (0.717) (0.595)	-0.83 [-14.15-12.48] ^a^ -2.48 [-16.21-11.24] ^b^ -3.78 [-18.07-10.51] ^c^	-0.03 [-0.61 to 0.55] ^a^ -0.09 [-0.67 to 0.49] ^b^ -0.14 [-0.72 to 0.44] ^c^
P-ER	52.65 (15.02)	74.52 (10.63)	< 0.001	55.09 (19.29)	76.26 (18.57)	< 0.001	(0.940) (0.994) (0.865)	0.23 [-6.1-6.57] ^a^ -0.02 [-6.6-6.5] ^b^ -0.58 [-7.45-6.29] ^c^	-0.01 [-0.56 to 0.59] ^a^ -0.001 [-0.58 to 0.58] ^b^ -0.04 [-0.62 to 0.54] ^c^
P-IR	51.09 (9.33)	70.22 (11.9)	< 0.001	53.57 (12.14)	70.7 (13.91)	< 0.001	(0.863) (0.867) (0.757)	-0.62 [-7.9-6.65] ^a^ -0.62 [-8.18-6.93] ^b^ -1.21 [-9.09-6.66] ^c^	-0.05 [-0.62 to 0.53] ^a^ -0.05 [-0.63 to 0.53] ^b^ -0.09 [-0.67 to 0.48] ^c^
S-FLX	1.77 (1.04)	2.95 (1.2)	< 0.001	1.88 (0.82)	2.91 (1.05)	< 0.001	(0.518) (0.482) (0.647)	-0.13 [-0.56- 0.28] ^a^ -0.15 [-0.6-0.29] ^b^ -0.1 [-0.57-0.36] ^c^	-0.03 [-0.63 to 0.56] ^a^ -0.05 [-0.64 to 0.54] ^b^ -0.004 [-0.59 to 0.59] ^c^
S-ABD	1.63 (0.95)	2.5 (1.13)	< 0.001	1.66 (0.76)	2.47 (0.94)	< 0.001	(0.651) (0.635) (0.869)	-0.08 [-0.48-0.3] ^a^ -0.09 [-0.51-0.31] ^b^ -0.03 [-0.46-0.39] ^c^	-0.004 [-0.6 to 0.59] ^a^ -0.02 [-0.61 to 0.57] ^b^ 0.05 [-54 to 0.64] ^c^
SPADI	74.86 (20.63)	31.86 (18.91)	< 0.001	68.43 (20.56)	33.73 (23.92)	< 0.001	(0.186) (0.111) (0.138)	6.42 [-3.23-16.09] ^a^ 8.02 [-1.92-17.97] ^b^ 7.97 [-2.63-18.23] ^c^	0.3 [-0.28 to 0.89] ^a^ 0.38 [-0.2 to 0.97] ^b^ 0.37 [-0.21 to 0.95] ^c^

NPRS, numeric pain rating scale; ROM, range of motion; SPADI, shoulder pain and disability index; A-FLX, active flexion; A-ABD, active abduction; A-ER, active external rotation; A-IR, active internal rotation; P-FLX, passive flexion; P-ABD, passive abduction; P-ER, passive external rotation; P-IR, passive internal rotation; S-FLX, strength of flexion; S-ABD, strength of abduction; MD, mean differences; SMD, standardized mean differences

* Data are mean (SD). The baseline and after values provide the result of participants’ assessment before and after 4 weeks

^**^ Analyzed using ANOVA/ANCOVA tests

^a^ Adjusted to pre-intervention values of the outcomes (as covariate)

^b^ Adjusted to pre-intervention values of the outcomes and age (as covariate)

^c^ Adjusted to pre-intervention values of the outcomes and age (as covariate) and gender (as factor)

### Adverse events

In this study, 29 patients (67.5%) experienced bleeding with a higher frequency in in the experimental group (16 cases) compared to the control group (13 cases). Additionally,17 patients (39.5%) experienced bruising with a higher frequency in the control group (11 cases) compared to the experimental group (6 cases). Furthermore, 26 patients (60.5%) reported pain during the needling procedure, with significantly more case in the experimental group (22 cases) compared to the control group (4 cases). 17 patients (39.5%) reported muscle soreness after dry needling, and 2 patients (4.6%) experienced drowsiness in the experimental group [27]. It should be noted that all reported adverse events were cleared up within 24–36 h. All of the mentioned minor adverse events were managed by the study’s principal physiotherapist (FN), who had also informed the participants about these potential adverse complications before the study (Table 5).

**Table 5:** Reported minor adverse events throughout the course of study in the MTrP-DN and S-DN group

Event	Number reported	Percentage per total treatments
Bleeding	29	67.5%
Bruising	17	39.5%
Pain during	26	60.5%
Pain after	17	39.5%
Aggravated symptoms	0	0%
Drowsiness	2	4.6%
Feeling faint	0	0%
Headache	0	0%
Nausea	0	0%

## Discussion

According to the findings of our study, implementing 4 sessions of MTrP-DN for shoulder girdle muscles in the MRh did not show statistically greater improvement in postoperative shoulder pain, ROM, strength, and SPADI than adding a sham intervention. The small effect sizes suggest that the overall impact of MTrP-DN may be relatively small. Further research is needed to explore the factors contributing to these findings and to evaluate the intervention’s effectiveness in a broader context.

Postoperative shoulder pain is one of the most prevalent complications following RCR surgery [4]. ROM impairments and loss of strength that may be due to postoperative shoulder pain, period of upper extremity immobilization in the abduction orthosis, MTrP developments, and associated neuro-motor abnormalities are further serious complications of this surgery [4, 5]. Activation of MTrPs following the surgery may aggravate symptoms and extend the time of recovery for the patients [5, 11, 13]. Accordingly, this trial was designed to investigate whether including a muscle treatment approach in the MRh would improve the patients’ shoulder pain and the associated ROM impairments and loss of strength. The findings of the current study regarding postoperative shoulder pain and reported ROMs were consistent with those of Arias-Buría et al.’s, which found no statistically significant difference between the MRh and MRh with a single session of MTrP-DN for patients with a history of RCR or those who had proximal humeral fracture treated with open reduction and internal fixation using a PHILOS plate [13]. Due to lack of standardization in MTrP-DN dosage [15] and also based on the findings of the Arias-Buría’s study, we decided to implement more sessions of MTrP-DN in the MRh to determine whether more sessions of MTrP-DN would reduce the patients’ postoperative shoulder pain [13, 15] The findings of our study were also consistent with those of Halle et al.’s, which found no superiority in adding 4 sessions of MTrP-DN to the standard rehabilitation care over the standard rehabilitation care alone for patients following shoulder stabilization surgery [14]. The participants of our study and the other two trials were patients who had undergone shoulder surgery and suffered from postoperative shoulder pain. However, the pathology and the characteristics of these studies varied, and the activation of MTrPs and the associated pain may be more probable in RCR because the target tissues in this kind of surgery are muscles and their related tendons.

In contrast to the results of our study, the findings of a systematic review by Lin Liu et al. on the use of MTrP-DN for MTrPs associated with neck and shoulder pain showed that utilizing MTrP-DN is superior to using S-DN in short-term and medium-term follow-ups [12]. Moreover, a controlled trial study by Yu Bin Pai et al. showed a larger pain intensity reduction in the MTrP-DN group compared to the S-DN group in patients with chronic shoulder pain [41]. A potential explanation for this contrast in results may be due to the selected S-DN technique in this study. The subcutaneous needling approach, which was used as a ‘‘participant blinding strategy” in the control group in the current study [29], may explain the non-significant results and “small” effect sizes for postoperative shoulder pain and the associated ROM impairments. Because inserting a needle into the skin similar to deep dry needling can stimulate Aδ nerve fibers, which leads to an increase in the activity of enkephalinergic inhibitory interneurons in the dorsal horn [42]. This latter activity, caused by needle-induced stimulation of Aδ fibers in the tissues overlying a MTrP, prevents the central transmission of noxious information produced in group IV sensory afferent nociceptors (C-fibers) of the MTrP [42]. Additionally, it is also plausible that the subcutaneous needling procedure, similar to deep dry needling, may improve microcirculation and decrease chemical mediators in the tissues overlying a MTrP. Experiencing bleeding and bruising in both groups [43] as adverse events may confirm the occurrence of these mechanisms in both groups. Consequently, these potential needle-induced mechanisms may help both the experimental and control groups to have less postoperative shoulder pain. In addition, the activation of these mechanisms may potentially have an impact on the ROM impairments following postoperative shoulder pain which could also explain the small effect sizes in the reported active and passive ROMs between groups. The findings of the study by Hoseininejad et al. regarding the differences between subcutaneous and deep dry needling showed that both of these techniques could be effective in reducing pain and disability in patients with active MTrPs. However, deep dry needling seems to be more effective for improving muscle function [44]. Another plausible explanation for the absence of additional benefits could be that the MTrP-DN technique used in this study may have acted on the same outcomes targeted by the exercise-based rehabilitation protocol. Indeed, based on the findings of the recent studies, therapeutic exercise has shown to be effective for muscle recruitment and the restoration of shoulder motor control. Therefore, in this case the therapeutic exercise protocols may have surpassed or masked the MTrP-DN’s effects [45, 46].

The findings provided by Arias-Buría et al. did not match the findings of our research in terms of strength and functional status since the participants in their study were patients who had experienced chronic postoperative shoulder pain for an average of 5 months following the surgery and their demographics were different from the participants of our study [13]. In addition, the dynamometry method and the chosen score for self-reported functionality in Arias-Buría’s study were different from ours (9). Consequently, in contrast to our research, Arias-Buría showed that adding a single session of MTrP-DN to the MRh was superior to the MRh alone for shoulder strength and function [13]. However, the results of our study support the findings of Halle et al. in the SPADI, and both of these investigations revealed no statistically significant difference for the inclusion of MTrP in the MRh for the patients following shoulder surgery [14]. Although our study showed a small effect size for SPADI.

This trial also reported several minor adverse events that were cleared up within 24 to 36 h and there were no further complaints by the patients since all adverse events were managed by the study’s principal physiotherapist (FN). Mild bleeding (67.5%) and bruising (39.5%) were the common adverse events which were difficult to prevent [47]. Mild pressure over the needling site with a cotton ball was sufficient to minimize bleeding [47]. It should be noted that all the participants were screened for bleeding disorders and the use of anticoagulants before the study began. Another common adverse events were pain during (60.5%) and pain after (39.5%) the needling procedure as a result of neuromuscular injury or hemorrhagic and inflammatory changes [47]. Post-needling pain was cleared up in less than 72 h. Drowsiness was another adverse event reported by two patients (4.6%) in the experimental group. Symptoms of drowsiness were managed by removing the needles and elevating the patients’ feet in the supine position [47]. In addition, their blood pressure and pulse rates were monitored for 24 h. Furthermore, the results revealed several key differences between the experimental and control groups. The experimental group had a higher frequency of bleeding and pain compared to the control group, which had a higher frequency of bruising. These imbalances suggest that the MTrP-DN might be associated with increased pain and bleeding, whereas the S-DN could be linked to more bruising. The reason for these differences warrant further investigation, as they could impact the overall assessment of the MTrP-DN’s efficacy and safety.

Moreover, based on the findings of our study, the deltoid, upper trapezius, and levator scapula were the most involved muscles with MTrPs. Accordingly, further studies need to investigate the relationship between the presence of active MTrPs in the mentioned muscles and postoperative shoulder pain following the RCR surgery.

Based on our knowledge, this randomized controlled trial was the first to evaluate the efficacy of 4 sessions of MTrP-DN added to the MRh for patients following RCR surgery over the course of 4 weeks. However, future trials may report different results by implementing more sessions of MTrP-DN with long-term follow-ups in the primary stages of postoperative shoulder pain. In addition, according to recent studies in the field of MTrP-DN for patients with a history of shoulder surgery, conducting a systematic review may assist researchers in obtaining more conclusive results on the aforementioned subject.

## Limitations

There were several limitations to the current research that should be addressed. First, we used the subcutaneous needling technique as a participant blinding strategy that may have impacted the results of our study by stimulating Aδ nerve fibers and increasing microcirculation which might have helped with the management of MTrP-induced pain in the control group. So, we highly recommend the use of non-penetrating blinding strategies in the control group for future controlled trials. Second, we only implemented 4 sessions of MTrP-DN and collected data after 4 weeks of intervention. We did not have follow up beyond 4 weeks or long-term follow-ups to determine the possibility of long-term differences between these two groups. Therefore, we also recommend long-term follow-ups with more sessions of MTrP-DN for postoperative shoulder pain. Third, inability to control concomitant treatments, including analgesics usage, in order to keep the study participants compliant was another limitation in our study. Fourth, enrollment from a single clinic that may have increased the possibility of contamination bias was another limitation. Therefore, we suggest the feasibility of multi-center investigations on MTrP-DN for the patients following RCR surgery. Fifth, in the current study, age, gender, and pre-intervention values of outcomes were considered as important covariates to be adjusted for analysis. Although, there are several other potential confounding factors including medication use, tear characteristics, and number of repaired tendons. We recommend considering these factors in future investigations. Sixth, we included individuals with a wide age range (40–75) to increase the generalizability of the study, although we recommend future studies implement subgroup analysis for age or design trials with narrower age ranges. Seventh, the results of this trial should be interpreted conservatively because it is preliminary in nature and the sample size is small.

## Conclusion

The results of our preliminary study indicate that including MTrP-DN of the shoulder girdle muscles in the MRh following RCR surgery did not yield statistically significant results for postoperative shoulder pain, active and passive ROM, strength, and SPADI compared to adding S-DN in the MRh at the end of 4 weeks of care.

In addition, it should be noted that using the subcutaneous needling technique as a participant blinding strategy in the S-DN group may have specific effects that mask the value of MTrP-DN. Consequently, further research with different control groups, larger sample sizes and more robust methodologies is warranted to better understand the MTrP-DN efficacy and to determine whether dry needling could be a valuable adjunct in the management of postoperative shoulder pain.

This study found that bleeding and pain during the needling procedure occurred more frequently in the experimental group compared to control group, while bruising was more common in the control group. these differences highlight the need to carefully consider these imbalances when evaluating the efficacy and safety of MTrP-DN. Future research should address these discrepancies to better understand their impact on patient outcomes.

## Data Availability

No datasets were generated or analysed during the current study.

## Abbreviations

MTrP: Myofascial trigger point
MTrP-DN: Myofascial trigger point dry needling
S-DN: Sham dry needling
MRh: Multimodal rehabilitation protocol
NPRS: Numeric Pain Rating Scale
SPADI: Shoulder Pain and Disability Index
ROM: Range of motion
RCT: Rotator cuff tear
RCR: Rotator cuff repair
ANCOVA: Analysis of covariance
MCID: Minimal Clinically Important Difference
ITT: Intention to treat
IPAQ-SF: International Physical Activity Questionnaire-Short Form
A-FLX: Active flexion
P-FLX: Passive flexion
A-ABD: Active abduction
P-ABD: Passive abduction
A-IR: Active internal rotation
P-IR: Passive internal rotation
A-ER: Active external rotation
P-ER: Passive external rotation
S-FLX: Strength of flexion
S-ABD: Strength of abduction
